# Studies on mosquito biting risk among migratory rice farmers in rural south-eastern Tanzania and development of a portable mosquito-proof hut

**DOI:** 10.1186/s12936-016-1616-8

**Published:** 2016-11-22

**Authors:** Johnson K. Swai, Marceline F. Finda, Edith P. Madumla, Godfrey F. Lingamba, Irene R. Moshi, Mohamed Y. Rafiq, Silas Majambere, Fredros O. Okumu

**Affiliations:** 1 Environmental Health and Ecological Sciences Thematic Group, Ifakara Health Institute, Ifkara, Tanzania; 2 School of Public Health, University of the Witwatersrand, Parktown, Johannesburg, Republic of South Africa; 3 Innovative Vector Control Consortium, Liverpool, UK

## Abstract

**Background:**

Subsistence rice farmers in south-eastern Tanzania are often migratory, spending weeks or months tending to crops in distant fields along the river valleys and living in improvised structures known as *Shamba* huts, not fully protected from mosquitoes. These farmers also experience poor access to organized preventive and curative services due to long distances. Mosquito biting exposure in these rice fields, relative to main village residences was assessed, then a portable mosquito-proof hut was developed and tested for protecting these migratory farmers.

**Methods:**

Pair-wise mosquito surveys were conducted in four villages in Ulanga district, south-eastern Tanzania in 20 randomly-selected *Shamba* huts located in the distant rice fields and in 20 matched houses within the main villages, to assess biting densities and *Plasmodium* infection rates. A portable mosquito-proof hut was designed and tested in semi-field and field settings against *Shamba* hut replicas, and actual *Shamba* huts. Also, semi-structured interviews were conducted, timed-participant observations, and focus-group discussions to assess experiences and behaviours of the farmers regarding mosquito-bites and the mosquito-proof huts.

**Results:**

There were equal numbers of mosquitoes in *Shamba* huts and main houses [RR (95% CI) 27 (25.1–31.2), and RR (95% CI) 30 (27.5–33.4)], respectively (P > 0.05). Huts having >1 occupant had more mosquitoes than those with just one occupant, regardless of site [RR (95% CI) 1.57 (1.30–1.9), P < 0.05]. Open eaves [RR (95% CI) 1.15 (1.08–1.23), P < 0.05] and absence of window shutters [RR (95% CI) 2.10 (1.91–2.31), P < 0.05] increased catches of malaria vectors. All *Anopheles* mosquitoes caught were negative for *Plasmodium*. Common night-time outdoor activities in the fields included cooking, eating, fetching water or firewood, washing dishes, bathing, and storytelling, mostly between 6 and 11 p.m., when mosquitoes were also biting most. The prototype hut provided 100% protection in semi-field and field settings, while blood-fed mosquitoes were recaptured in *Shamba* huts, even when occupants used permethrin-impregnated bed nets.

**Conclusion:**

Though equal numbers of mosquitoes were caught between main houses and normal *Shamba* huts, the higher proportions of blood-fed mosquitoes, reduced access to organized healthcare and reduced effectiveness of LLINs, may increase vulnerability of the itinerant farmers. The portable mosquito-proof hut offered sufficient protection against disease-transmitting mosquitoes. Such huts could be improved to expand protection for migratory farmers and possibly other disenfranchised communities.

## Background

Vector control, plays a central role in the fight against malaria and other mosquito-borne illnesses [1–3], and historical evidence suggests that well organized vector control operations can effectively achieve elimination in local areas [4–6]. Over the years, technological solutions including long-lasting insecticide treated nets (LLINs), indoor residual spraying (IRS), prompt diagnosis and treatment, as well as development of vaccines and new drugs have dominated the malaria control agenda, while novel environmental management strategies and improved housing, though effective [7, 8], have only been scantly considered.

To reduce malaria infections to zero, it will be essential to effectively identify and target the last remaining pockets of transmission, including geographically distinct areas of high transmission, but also demographically high-risk sub-populations, such as migratory forest workers and itinerant farmers. In subsequent phases of malaria control, such targeting will be required to ensure that there are no residual pockets of transmission or individuals who would act as reservoirs of transmission [9–11].

**Fig. 1:**
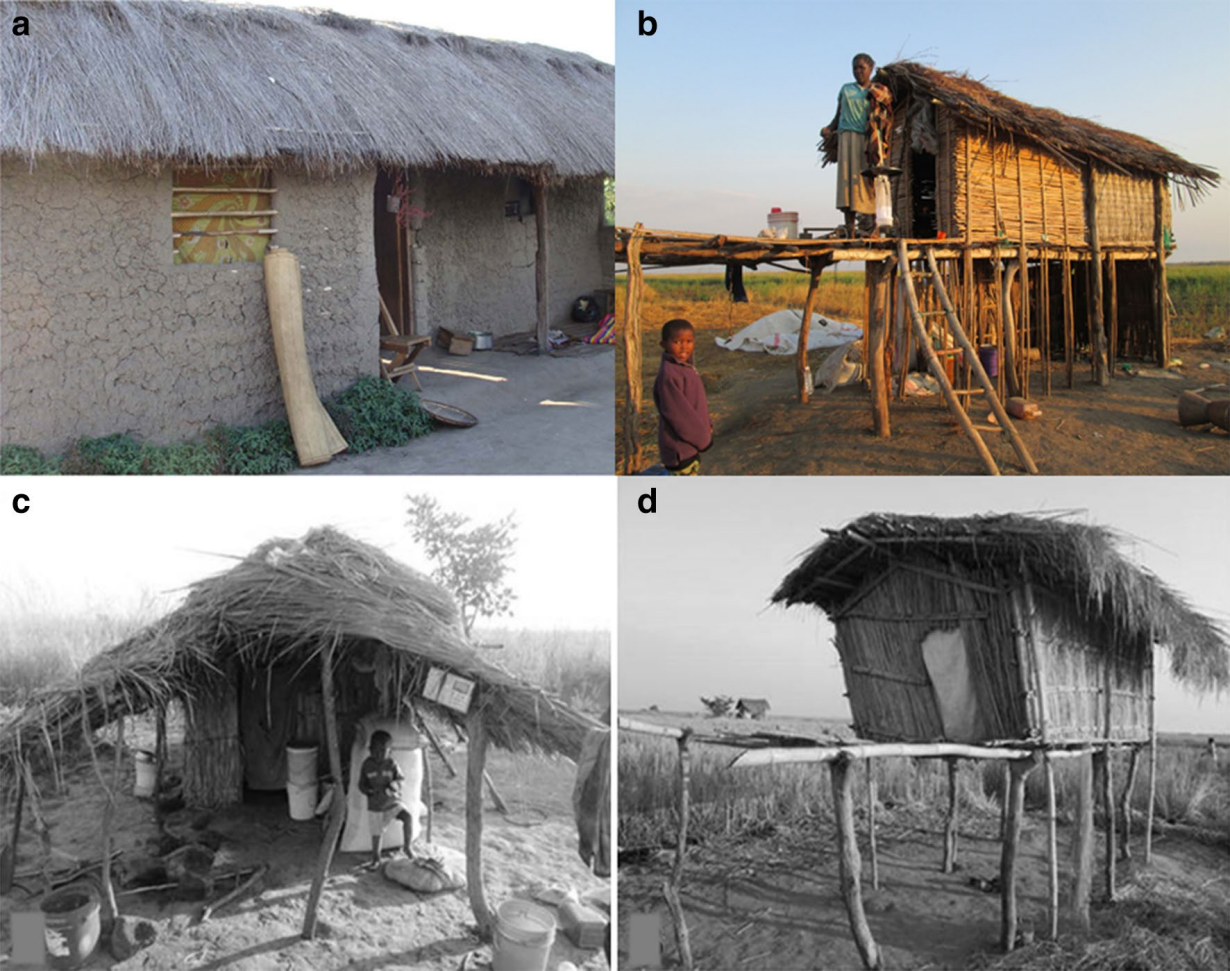
Residential homes and *Shamba* huts. Picture of a typical local house used in the main villages in rural south-eastern Tanzania (**a**), and typical *Shamba* huts used by migratory farmers when away in their distant rice fields (**b**–**d**)

In rural south-eastern Tanzania, where long-lasting insecticidal bed nets have been widely used, malaria prevalence has been reduced by >60% since 2001 [12], but low-level transmission still persists. Most rural households here practice subsistence migratory farming, where farmers regularly move to their distant rice fields, and spend weeks–months tending to their crops. Usually, these migratory families bring with them children below school-going age, including breast-feeding babies [13, 14]. Because of their migratory livelihoods, lack of proper protective measures against mosquito bites and reduced access to organized health care, the farming sub-population, just like forest workers [15, 16], could be a potent parasite reservoir, perpetually seeding parasite transmission upon return to the main villages. Yet, these sub-populations are often left out by existing conventional malaria prevention programmes [17]. While away at the farms, the families live in improvised, temporary, and semi-open shacks (locally referred to as *Shamba* huts), unprotected from mosquito bites and mosquito-borne illnesses (Fig. 1). Even where insecticide-treated bed nets (ITNs) are provided, the inability to properly mount nets in these improvised structures, often with no beds, leave the households only partially protected. Besides, other proven effective interventions such as IRS with insecticides cannot be readily implemented in these *Shamba* huts, which usually have no proper sprayable surfaces. As a result of exposure to mosquito-borne illnesses like severe malaria and anemia, these families may experience reduced productivity in their farms, significant loss of man-hours and loss of human lives.

There is a large body of data, from as early as beginning of the twentieth century showing that screening and modifying house structures can protect people from malaria and other mosquito-borne illnesses [18]. In recent years, greater evidence has been obtained that demonstrates effectiveness of improved housing as a significant barrier to vector borne diseases [8], rejuvenating the efforts to pursue this strategy. For migratory communities such as the farmers in rural south-eastern Tanzania, improved housing conditions would also allow more effective use of proven interventions, such as LLINs and IRS, which are otherwise not readily usable inside the current *Shamba* huts.

This study comparatively assessed nightly mosquito-biting and *Plasmodium* infection risk experienced by migratory rice farmers in Ulanga district, south eastern Tanzania, while they are in the fields or in their main villages. A portable mosquito-proof hut was then developed and tested for protecting these farmers while in their distant fields.

## Methods

### Study area

**Fig. 2:**
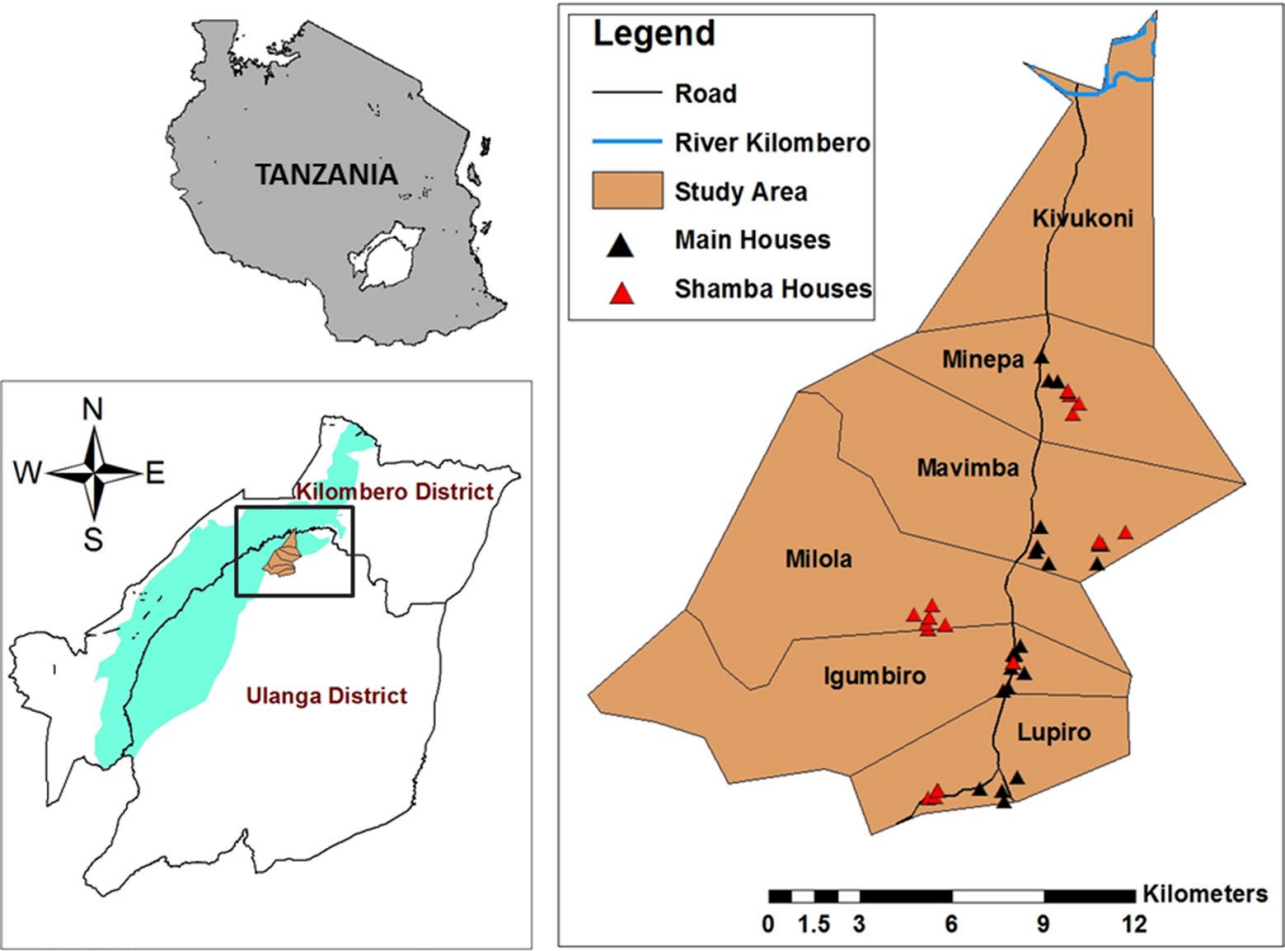
Map of study area. Map showing study area, including the four villages in Ulanga district, south-eastern Tanzania, where the study was done

The study was conducted in Ulanga District, Morogoro region, Tanzania, in the villages of Minepa, Mavimba, Igumbiro and Lupiro, along the Kilombero river valley (Fig. 2). The climate is hot and humid, with an annual rainfall between 1200 and 1800 mm and mean annual temperature of 20–32 °C [19]. All the villages have moderate perennial malaria transmission [20], as climatic conditions and rice farming (both irrigated and non-irrigated) create ideal conditions for high densities of mosquitoes [21]. Most community members are subsistence farmers, cultivating mostly rice, but also maize and other crops such as sweet potatoes and beans. The study included both permanent household residences in the villages, and in the distant semi-open improvised farm houses, commonly referred to in Kiswahili language as *Shamba* huts (Farm huts), where many adults spend significant periods of time tending to their crops. According to data from the Ifakara Health Institute Health and Demographic Surveillance System (HDSS), the local houses in the main villages have walls mostly made up of mud (56%), or of baked mud bricks [19]. The roofs are mostly thatched (70%) or of corrugated iron sheets [19] (Fig. 1a). The temporary structures (hereinafter referred to as *Shamba* huts) that are used by the migratory farmers in the fields are made from bamboo stems; sometimes have thatched grass/palm tree leaves for walls or just mud. Some are raised on stilts for protection from water and wild animals [22], and to give the farmers vantage when watching over their crops (Fig. 1b). The evaluation of the prototype mosquito-proof hut was done inside semi-field systems (SFMs) at Ifakara Health Institute, Kining’ina campus (8.11417°S, 36.67484°E). Details of the design and use of this SFS have been provided previously [23, 24].

### Entomological assessments of human-biting mosquito densities inside and around the houses used by residents while in the main villages, and *Shamba* huts used while in the rice fields

First, enumeration of all the active *Shamba* huts in areas surrounding the four villages, Minepa, Mavimba, Igumbiro and Lupiro villages of Ulanga district, at the beginning of the study period. A full listing of main houses in the same villages was also obtained from Ifakara Health Institute HDSS. From the master list of *Shamba* huts, five *Shamba* huts were randomly selected in each village, so that there were 20 selected *Shamba* huts, located at the edges of the 4 different villages. To match the twenty *Shamba* huts used when the farmers are out in their farms, a set of 20 main houses regularly used by families were selected in the same four villages. The *Shamba* huts were matched village wise to the main houses, such that the *Shamba* huts were located in the adjacent rice fields near each of the villages. This way, in each village, a set of five main houses was paired with a set of five *Shamba* huts. These surveys were initially done in July and August 2013 and then repeated between July and September 2014. To quantify actual biting exposure in the *Shamba* huts relative to biting exposure within the main villages, mosquito collections were conducted in the selected main houses and also in the *Shamba* huts located at the edge of each of these respective villages.

Indoor collections were done using Centre for Disease Control (CDC) light traps^®^ set next to occupied bed with a person under a bed net [25, 26], from 1830 to 0700 hours each night, while outdoor collections were done using a newly-designed exposure-free system for conducting human-baited catches, where an adult male volunteer sits inside a two-chambered netting cage and catches mosquitoes before they actually reach the volunteer [27]. In this system, also called the M-Trap and earlier described by Mwangungulu et al. [27], the volunteer can sit during the night protected from mosquito bites, and mosquitoes attempting to bite him are trapped within the second compartment also having netting walls. Mosquitoes enter the system through three envelope-shaped entry points on the sides. Five such outdoor collection stations, each with an adult male volunteer (18–35 years old) were set up near the same five *Shamba* huts and another five M-traps set up near the matching main houses in the main villages. During these mosquito collections, continuous observations of temperatures and humidity were also done on hourly basis, inside both the *Shamba* huts and the main houses using portable indoor climate Tinytag Plus^®^ data loggers (Omni Instruments, London, UK).

### Design, construction and testing of a prototype mosquito-proof hut for use by the migratory rice farmers while away in their distant rice fields

#### Design

**Fig. 3:**
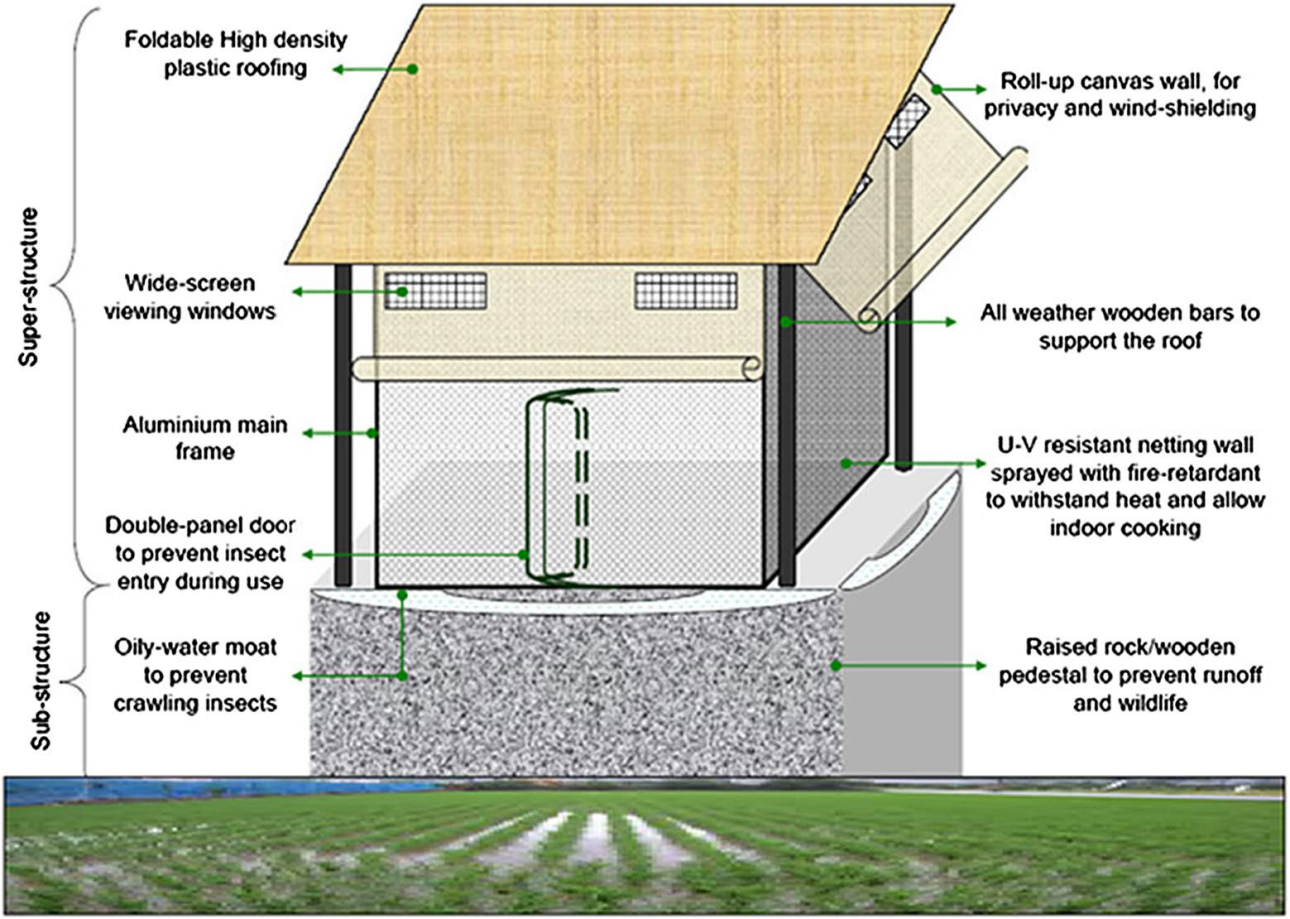
Labelled schematic of proposed portable mosquito-proof hut. The design of the portable mosquito-proof huts (the *Swai* huts)

**Fig. 4:**
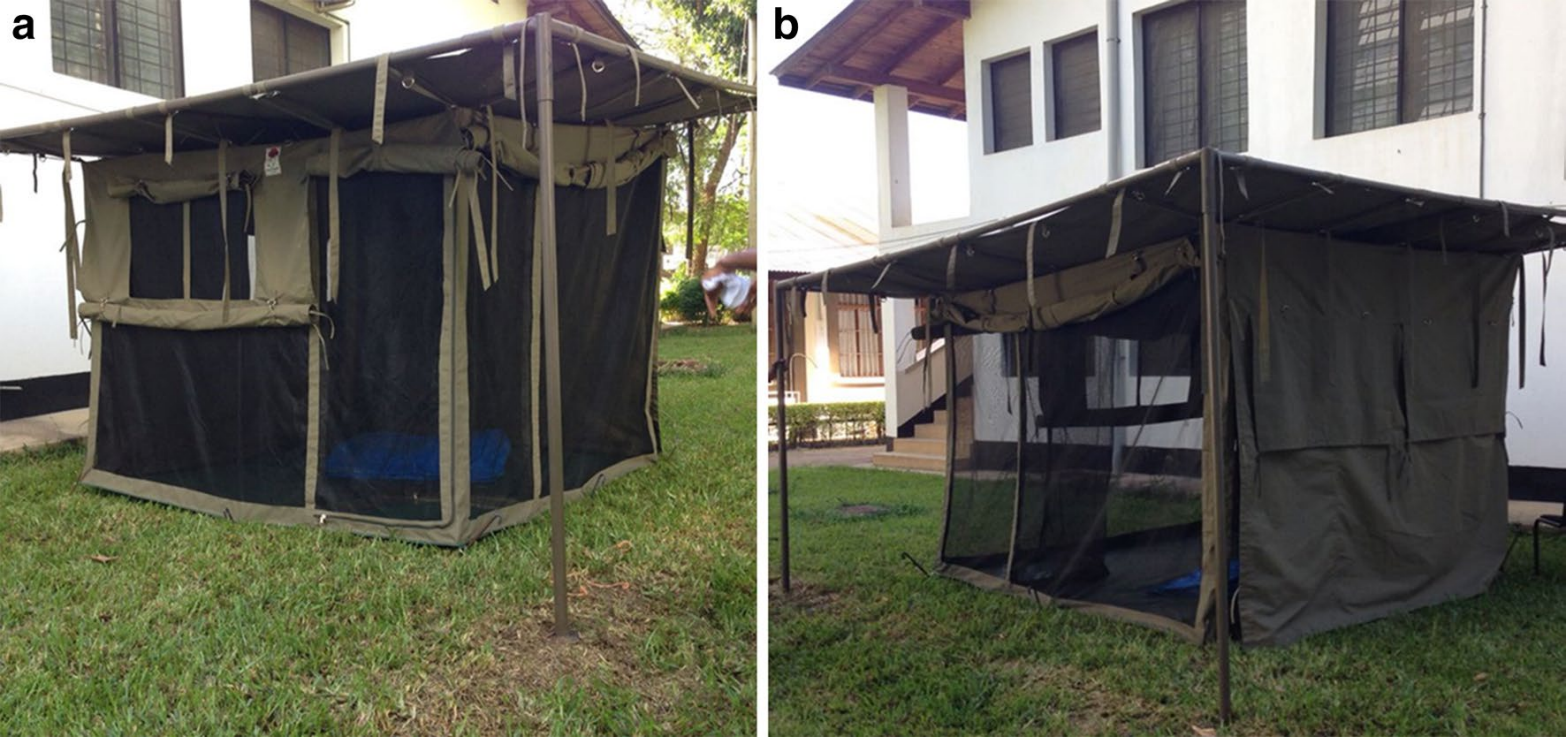
*Swai* hut prototype. Picture showing the front (**a**) and side (**b**) views of the *Swai* hut prototype

The main aim was to create a portable mosquito-proof hut prototype with the following essential characteristics: (1) easy to transport, (2) large enough to accommodate a migratory family of two adults and one child, (3) easy for one person to set up while in the field on his/her own, (4) robust and durable for long-term field use, (6) highly ventilated and (7) can be mounted on basic pedestals already being used by farmers in the study area (Fig. 1b, d). A tentative hut design to meet these features was proposed (Fig. 3), upon which the structural engineers at the partnering company (Elastic Product Manufacturing Company Limited, Tanzania), worked to create the final prototype. Construction was done based primarily on this original design, while also considering preferences suggested by the farmers during our interviews and focus-group discussions, as well as additional modifications from the expert engineers. The final prototype design, also called *Swai hut* is shown in Fig. 4.

The basic structure consists of a 10ft × 10ft × 8ft steel frame supporting an 8ft × 8ft × 8ft square housing structure made of durable canvas and UV-resistant shade netting. It has large windows on the sides, with foldable canvas window flaps that can be rolled up or down to close the windows, and/or the entire side walls of the huts. It has wide screen viewing windows, which also improve ventilation and air flow. The large windows and open netting structure ensures utmost ventilation in the huts. The inside surface has a separating canvas wall that can be rolled up or down depending on need. The floor of the hut is made of thick poly-vinyl chloride (PVC) canvas, which is water proof, and extends upwards on the sidewalls forming a water-proof skirting for added protection. The roofing is designed to slightly slant backwards so that whenever it rains, all the rain water easily flow backwards, without seeping into the huts. This roofing material is foldable and made of high density polyethylene material. To enhance protection from biting insects, the huts have a double-panel door to prevent insects. The hut is fitted with hooks on the sides attached to the steel beams so that it can be tightly secured onto the ground, or mounted on top of a pre-fabricated sub-structure, as is common practice in rural-south eastern Tanzania (Fig. 1b, d). All the doors are secured using high-strength zippers, while the roll-down canvases, over the windows have laces so that they can be tightly fastened. This initial prototype was made at a total cost of US$ 1460.38 inclusive of construction labour and value added tax.

### Semi-field and field testing of the portable huts to assess protection from host-seeking disease-transmitting mosquitoes

Controlled semi-field and field experiments were conducted to demonstrate that the portable mosquito-proof house can reduce mosquito house entry and bites. The semi-field experiments were conducted inside two chambers of the SFS. Each of the semi-field chambers used measured 9.6 m × 9.6 m, inside which there was growing vegetation, thus mimicking real-life mosquito ecosystems and villages [23].

The portable mosquito proof prototype was assembled in one of the chambers and a locally-made *Shamba* hut replica (of similar characteristics to those described and seen in the rice fields, but with dimensions similar to the prototype) was constructed in a different chamber of same size, so that there was a treatment and control chamber. A pair of consenting male volunteers were recruited to sleep inside each of the houses under bed nets as basic protection. Each night, 500 hungry 6–8 days old laboratory-reared female *Anopheles arabiensis* mosquitoes that had not previously taken any blood meals were released into the semi-field chambers, 1 h before start time of the experiments, which was 1900 hours. In the first round of experiments, the volunteers were provided with intact new Olyset^®^ nets, while in the second round they were provided with bed nets having 20 holes measuring 2 cm × 2 cm to mimic torn nets. The test was done for two rounds, each lasting 10 days. The different hut types were rotated between the two chambers, in a 2 × 2 cross-over design while the volunteers and hut positions remained fixed. Mosquito collections in both *Swai* hut prototype and the *Shamba* hut replica was done throughout the night using CDC light traps^®^ set next to the volunteer-occupied bed net inside the huts [25, 26]. Each morning, any mosquitoes left resting or dead on the walls, floor and other surfaces of the two huts were also collected by the volunteers, in this case using mouth aspirators.

Full field experiments were conducted in 100 m × 100 m open field sites in each of the four study villages in Ulanga district, south eastern Tanzania. In each of the villages, the portable mosquito proof hut and a replica *Shamba* hut (similar to the one used in semi field experiments) were placed 50 m away from each other and compared directly. A pair of consenting adult male volunteers was recruited to sleep inside each of the huts under Olyset^®^ nets each night. This was done for 16 days in each of the four villages, with the two hut types rotating positions on the ninth day, to account for any positional bias. The volunteers however did not change their positions, and in this way, the volunteers and position were taken as a single source of experimental variation, as the hut types were rotated. Mosquito collections inside both the *Swai* hut prototype and the *Shamba* hut replica were done throughout the night using CDC light traps^®^ set next to the occupied bed net [25, 26]. Each morning, any mosquitoes resting or dead on the walls, floor and other surfaces of the two huts were also collected by the volunteers using mouth aspirators. These binary 16-night comparative tests were repeated in each of the four villages, working with a different pair of volunteers per village.

After the field controlled trials, the *Swai* hut design was tested when in use with actual rice farming families as compared to normal *Shamba* huts that are used in the rice fields. This was done by rotating the *Swai* hut between four rice farming families in a 4 × 4 Latin square after every 10 days. The end of this final experiment coincided with the end of harvest season, when rice farmers were leaving the rice farms, back to the main villages.

### Mosquito identification

All the mosquitoes collected during the field experiments were sorted by taxa and blood feeding status (i.e. as blood fed, gravid or non-blood fed). The sorting was done on fresh samples each morning, without letting the mosquitoes dry. A sub-sample of *Anopheles gambiae s.l* and *Anopheles funestus* group mosquitoes was stored in small micro-centrifuge tubes (Eppendorf^®^), containing silica gel. These samples were further identified into sibling species through polymerase chain reaction (PCR) [28, 29]. Enzyme-linked immunosorbent assays (ELISA) were also conducted to determine *Plasmodium falciparum* sporozoite infection rates in the mosquitoes [30]. All the laboratory analysis were conducted at Ifakara Health Institute, Tanzania.

### Assessments of views, behaviours and experiences of the migratory rice farmers regarding malaria transmission and its control

A qualitative survey was conducted in the same four villages, Minepa, Mavimba, Igumbiro and Lupiro, where entomological surveys were done. This involved a stage-wise approach where three different complementary behavioural science methods for data collection were used, that is: (a) semi structured interviews (SSI) with household heads, (b) timed participant observations (PO) of activities conducted by members of households, and (c) focus group discussions (FGDs) with a selection of the community members who had participated in the SSI and PO assessments. All of these were implemented using study guides prepared and piloted in advance of the study.

A cross section of migratory rice farmers was identified using the non-probability sampling technique of snowballing among target populations in the study villages. This way the migratory farming households helped nominate others who were also migratory. Initially, the study team identified and planned to visit a total of 138 households (35–36 households per village), but this was reduced by half to 64 households (16 households per village), after the pilot study suggested a high level of homogeneity among the migratory farming households, who were giving highly similar answers indicating the data would be quickly saturated (i.e. answers from participants starting to be repetitive). During the SSIs, the researcher asked and gently probed for participants’ opinions on issues, such as: (a) whether they were aware of differences in risk of mosquito bites while in the rice fields compared to main villages, (b) whether they had any experiences with mosquito-borne diseases, including malaria, (c) what control or protective measures they were using while away in their farms, and (d) how they cope with bites and malaria infection whenever they are in the rice fields.

After, half of the interview candidates in each village (eight households per village) were then selected to participate in the timed participant observations to identify the main activities in which the migratory farmers and their family members were usually involved in at different times of the night, and which could expose them to mosquito bites. Selection of candidates for the participant observations was based on willingness to participate, as well as the presence of at least one member of the household who is able to read and write, so that he or she could conduct the actual observations after being trained. All activities carried out from 1800 to 0700 hours were catalogued in the observational checklist given to the trained family members in each participating household. This was done for three nights in each household, resulting in a total of 24 household-level observations in each of the four villages. The reason for relying on trained community members was the needed to minimize the observer bias, at times also referred to as the Hawthorne effect, where study subjects might change or modify their behaviours in response to being observed [31]. Every hour, the observers noted down by ticking a pre-defined check box whether any of the family members was participating in any of the stated outdoor activities. In case there was an activity being conducted, that had not been pre-included in the observation list, the observer wrote this down as well at the end of the observation sheet. This procedure allowed us to catalogue all outdoor human activities occurring in the peri-domestic space and to specify on hourly basis when each of these activities was most frequently done.

After the semi-structured interviews and timed-participant observations, a group of participants was recruited from each of these same villages to participate in FGDs on the observed outdoor behaviours and associated risks experienced in the rice fields and also the main villages. The FGD consisted of groups of 6–8 adults from the migratory farming communities. During these sessions, how the participants reacted to and interacted with the newly created *Swai* huts for protecting the migratory farmers was also assessed. These interactions with the *Swai* hut were also video-taped after group consent. Two FGDs were conducted in each of the four villages, males and females separately but with mixed ages ranging from 21 to 68 year olds. At the start of the first sessions of each FGD, the participants with help from the research team assembled the *Swai* hut prototype. The rest of the discussions were then conducted around the hut, while the participants handled the device, creating an opportunity for them to make direct suggestions on specific features that could or should be improved. A total of eight FGD’s were completed, during which a group of 6–8 adults participated in setting up the prototype hut, while discussing its potential benefits and limitations, focusing particularly on the mosquito-proof features, portable nature and ease-of-use. Each discussion lasted about 35–40 min excluding the assembly of the *Swai* hut prototype. These were conducted at school grounds in each of the villages. The other themes for the FGDs included key concerns and proposed coping strategies currently being used by migratory rice farmers while in the fields, considerations of housing as a protective measure against infections, and specific views on the portable mosquito-proof hut prototype i.e. the *Swai* hut.

### Data analysis

All quantitative data was entered and verified in Microsoft Excel 2010, after which analysis of the mosquito catches was performed using the open source R statistical software [32]. Relationships between the indoor mosquito densities and the different hut types were i.e. main houses, the *Swai* huts or the *Shamba* huts, were examined using generalized linear mixed effects models (GLMMs), with lme4 package [33]. Mosquito densities were modelled as a function of fixed factors including, house type and village, treating volunteer pairs and date of collection as random factors. To address the over-dispersion observed in the field data, a negative binomial family of models with log-link function was used.

The qualitative data on the other hand was analysed as follows: All audio formats of the SSI and FGD’s were transcribed and then translated from Kiswahili (the language in which the data had been collected) to English. The translated transcripts were then imported to *Atlas.ti* software and analysed as per the following themes: challenges in the distant farms, malaria prevention in the farms, effectiveness of traditional huts in preventing mosquito entrance and views regarding the newly designed portable mosquito-proof huts. A code book to allow easy identification of the different themes of interest from the translated transcripts was created. The observational data was entered into Epi Data^®^ software version 3.1 and then imported to STATA statistical analysis software package 9 (Stata Corp). All the different activities performed were tabulated with respect to time of night, and then the final histograms produced in Microsoft Excel.

## Results

### Mosquito catches in *Shamba* huts and main houses

In the initial surveys, comparing indoor mosquito densities between the *Shamba* huts used by migratory rice farmers while away in their distant field sites and mosquito densities in their main village houses, a total 22,959 female mosquitoes were caught. These included 7764 *An. gambiae s.l.* (all of which were later confirmed by PCR as *An. arabiensis*), 3262 *An. funestus*, 9618 *Culex* species mosquitoes, 2050 *Mansonia* species mosquitoes, and 5 *Aedes* species mosquitoes. All *Anopheles* mosquitoes caught were tested by ELISA for circumsporozoite *Plasmodium* proteins, but none tested positive.

On average, there was equal number of female mosquitoes in *Shamba* huts and main houses [RR (95% CI) 27 (25.1–31.2), and RR (95% CI) 30 (27.5–33.4)], respectively. However, huts having more than one occupant had more mosquitoes than those with just one occupant, regardless of whether it was in the rice fields or main villages [RR (95% CI) 1.57 (1.30–1.9), P < 0.05]. Open eaves [RR (95% CI) 1.15 (1.08–1.23), P < 0.05] and absence of window shutters [RR (95% CI) 2.10 (1.91–2.31), P < 0.05] increased catches of malaria vectors inside the huts. The temperature and humidity in the main houses and *Shamba* huts were almost similar with difference of <10 between them (Table 1).

**Table 1:** Lowest, mid and highest temperature in degree celsius and humidity in percentage recorded indoors of *Shamba* house replicas or the real *Shamba* houses, main houses and *Swai* hut

Location	Temperature (°C)			Humidity (%)		
	Lowest	Median	Highest	Lowest	Median	Highest
*Shamba* huts	18.6	25.9	38.6	30.4	68.2	98.3
Main houses	20.1	25.8	32.4	20.3	60.7	91.5
*Swai* hut	17.4	29.2	54.8	0.0	64.1	100.0

### Efficacy of the Swai hut prototype relative to the *Shamba* huts in semi-field and field settings

In both the controlled tests in the semi-field and full field, no mosquitoes entered the *Swai* huts, indicating 100% protection from potentially disease-transmitting mosquitoes. In the semi field tests where volunteers inside the respective huts used either intact or artificially holed nets (with 20 holes each measuring 2 cm × 2 cm); no blood fed mosquitoes were found in the *Swai* hut. Regarding protection from actual mosquito bites, it was observed that where the volunteers slept in the *Shamba* hut replicas, average number of blood fed mosquitoes found was 11.7 (6.7–16.7), when using holed nets and 0.4 (0–0.4) when using intact nets (Table 2). Similarly, in our field experiments, the prototype *Swai* hut completely prevented mosquito entry unlike in the replica *Shamba* huts, where substantial numbers of mosquitoes of different species were caught (Table 3). The *Swai* hut had similar ranges of temperature and humidity as both the main houses and *Shamba* huts except for the highest temperature reached (Table 3).

**Table 2:** Mean number of *Anopheles arabiensis* mosquitoes collected inside the *Swai* huts and the *Shamba* house replicas during the semi-field experiments

House type	Mosquitoes caught using CDC light traps		Mosquitoes collected resting on hut walls		Mosquitoes collected on the floor of the huts		Mosquitoes collected inside the bed nets	
	Mean no. unfed [LCI–UCI]	Mean no. blood-fed [LCI–UCI]	Mean no. unfed [LCI–UCI]	Mean no. blood-fed [LCI–UCI]	Mean no. unfed [LCI–UCI]	Mean No. blood-fed [LCI–UCI]	Mean No. unfed [LCI–UCI]	Mean No. blood-fed [LCI–UCI]
Tests with intact bed nets								
*Swai* hut	0 [0–0]	0 [0–0]	0 [0–0]	0 [0–0]	0 [0–0]	0 [0–0]	0 [0–0]	0 [0–0]
*Shamba* house replica	126.8 [61.8–191.8]	0 [0–0]	21.8 [15.8–27.8]	0 [0–0]	10.9 [5.9–15.9]	0 [0–0]	0 [0–0]	0.4 [0–0.4]
Tests with torn bed nets								
*Swai* hut	0 [0–0]	0 [0–0]	0 [0–0]	0 [0–0]	0 [0–0]	0 [0–0]	0 [0–0]	0 [0–0]
*Shamba* house replica	177.8 [137.8–217.8]	0 [0–0]	34 [24–44]	0.8 [0–0.8]	17.9 [8.9–26.9]	0 [0–0]	0 [0–0]	11.7 [6.7 –16.7]

**Table 3:** Mean number of mosquitoes of different taxa, collected inside the *Swai* huts, *Shamba* house replicas or the real *Shamba* houses during the field experiments in the four villages

House type	*A. gambiae* Mean [LCI–UCI]	*A. funestus* Mean [LCI–UCI]	*Culex* species Mean [LCI–UCI]	*Mansonia* species Mean [LCI–UCI]
Field tests against *Shamba* hut replicas				
*Swai* hut protoype (N = 16)	0 [0–0]	0 [0–0]	0 [0–0]	0 [0–0]
*Shamba* house replicas (N = 16)	30.2 [18.2–42.2]	2.9 [1.9–3.9]	11.8 [5.8–17.8]	59.8 [36.8–87.8]
Field tests against actual *Shamba* huts				
*Swai* hut prototype (N = 30)	0.3 [0.1–0.7]	0.4 [0.0–0.8]	0.1 [0.0–0.2]	0.1 [0–0.3]
*Shamba* houses (N = 90)	3.2 [2–4.5]	7.2 [5.3–9.1]	1.6 [1.0–2.1]	0.3 [0.0–0.6]

### Views and opinions of migratory farming households on mosquito biting risk, malaria transmission, and protection methods

Nearly all (96%) of the rice farming household heads interviewed knew that malaria infections were a result of being bitten by mosquitoes. It was found there are three distinct types of the itinerancy in the farming practices as follows: (a) 68% were those who relocated for extended periods with their whole family (including infants 1–6 months old) to the rice fields for the whole farming season, (b) 16% were those who relocated to their rice fields for the period of harvesting only, and (c) 16% were those who moved frequently between their main houses in villages and their rice fields, spending approximately 1–3 weeks in their rice fields. The study considered families spending <1 week at a time in their rice field as being non-migratory. Generally, the migratory rice farmers mostly spent more time in the rice fields for purposes of clearing weeds, protecting their rice from wild animals and harvesting time. Here are two direct quotes with examples of responses from study participants, when asked how long they stayed at their distant farms without returning home:

- I always stay for six months, (51 years old male, Igumbiro village).
- I shift to the farms for a week to three. When my work is done I return home, and when it is the time to weed I shift to the rice fields again to clear the weeds and return back home when am done, (22 years old female, Igumbiro village).

All of the migratory farmers used bed nets inside the *Shamba* huts while in the rice fields, even though these nets were not always perfectly fitting onto the sleeping spaces. Some of these bed nets had been received from ongoing net distribution campaigns in the main villages, but the families transferred the nets with them to the rice fields. Other protection methods used in the rice fields included, topical repellents, sitting next to a fire, and fanning the body with a piece of cloth so as to prevent mosquito bites. These other protection measures were mostly used outdoors. Here are examples of direct responses from the study participants, when asked how they protected themselves from mosquito bites during their stay at their distant farms:

- I use the bed net that we were given as aid. I usually sleep with my child and his mother (56 year old male, Lupiro village)
- When I am outside, it is mostly time to talk and if they increase (mosquitoes) you can take firewood with smoke. It helps a little. After we eat we go inside (28 years old male, Minepa village)
- The net helps when I go to sleep and chasing them away using hands or clothes when I am cooking. So I prefer both (50 years old female, Minepa).

### Outdoor activities of migratory farming households that may expose people to potentially infectious mosquito bites

A large number of the farmers said that they go to bed anytime between 2000 and 2300 hours while in the rice fields. During our direct observations of peri-domestic outdoor activities, it was also observed that most of the farmers were seen to go indoors between 2000 and 2300 hours, after which there was lower frequency of outdoor activities. Generally, farmers went indoors earlier (starting 2000 hours) when they were in the rice fields, in the main villages (starting 2100 hours), because of mosquito bites.

- …because I am not under a net, I am just outside cooking and eating while the mosquitoes are biting me. This is why I see it is better I go into the net early and rest because the more I sit outside the more the mosquitos bite me (46 year old female, Lupiro).

**Fig. 5:**
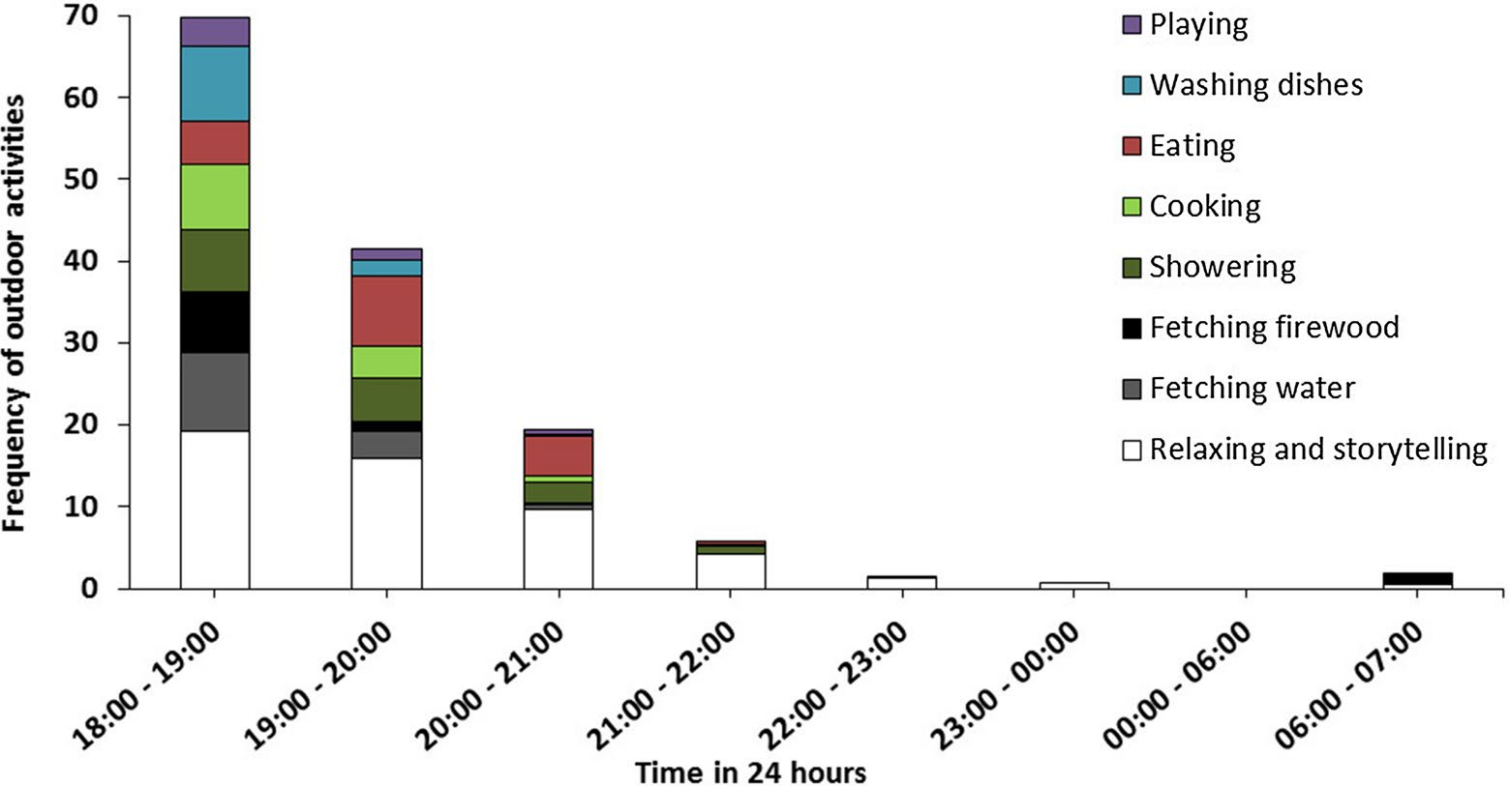
Outdoor activities done by adult migratory farmers from 1800 to 0700 hours. Frequencies of common outdoor activities performed by adult migratory farmers at different times of night while away in their distant rice fields

It is probable that they actually went to bed right after going indoors. From these observations, it was seen that the most common outdoor activities in the rice fields included relaxing and storytelling, cooking, eating, fetching water or firewood, washing dishes, playing and showering (Fig. 5).

### Responses of migratory farmers regarding the prototype mosquito-proof huts, i.e. Swai design

The participants were evidently pleased with the portable huts. They said the huts were attractive, well-ventilated and had a large sleeping area, and having extra netting meant that they (participants) would not even need to deal with mosquito nets. Regarding reconstruction of the portable huts, the participants noted that it was easy to follow the instructions, that it would not take much time to put up (requiring <1 h) when compared to building a traditional *Shamba* hut, which would require up to a week or more to complete. The only limitation that the participants voiced out was that the hut protected them only when they were inside and that while outside they would still unprotected. Below are some comments from the participants regarding the prototype hut.

- Honestly speaking, I am totally impressed by its appearance and durability. This will help me work comfortably without being disturbed by the mosquitoes, (34 year old female, Lupiro village).
- The hut provides a comfortable shelter, much like that of a house, it has big windows and doors hence can protect us from any danger, (43 year old female, Lupiro village).
- The size, the floor, the extra net and the windows ensure a constant passage of oxygen, (40 year old male, Lupiro village).

### Amount of money the farmers were willing to pay for the prototype mosquito-proof huts (Swai hut)

When asked how much they were willing to contribute for the portable huts, more of the answers varied, between Tshs 50,000 and 100,000 (US$ 22.86 and $45.72) with a few going up to Tshs. 200,000 (US$ 91.44). Here are some answers from male respondents on the price they are willing to pay

- R21: Tshs. 80, 000 ($36.58), (40 year old male, Igumbiro village)
- R43: Tshs 100, 000 ($45.78), (45 year old male, Lupiro village)
- R2: If I am told to contribute Tshs. 100,000 ($45.74) or even Tshs. 150,000 ($68.58). I will be ready because I will save every year’s building cost and I will use it for five years, (36 year old male, Mavimba village).
- R3: I can contribute Tshs. 200,000 ($91.44) (38 year old male, Mavimba village).

When the farmers were asked whether they are willing to exchange their produce for the hut, these are some of the responses they gave:

- R2: Yes I will, 36 year old male, Mavimba village).
- R3: … and even told to exchange with crops I will be ready. I really want the portable hut and I will exchange crops equal to the price intended, (38 year old male, Mavimba village).
- R47: …and I am also ready to exchange with my crops as per the cost of the hut at that moment, (43 year old male, Lupiro village).

### Farming schedules

The participants pointed out that the time of year when they went to the farms varied. Some farmers started moving to the farms as early as November every year, just before the planting season, and stayed through July, or as late as August, when harvesting was complete, in between returning to the main villages only intermittently for very short periods. The farmers argued that they stayed for long in the farms so that they can reduce the disturbances of moving to and fro the farms frequently and to also tend to the crops.

### Challenges in the farms and malaria prevention while there

The participants mentioned many challenges they faced while living in the farms. These included wild animals, conflict with other tribes, fire accidents, unsafe drinking water and diseases like dysentery and malaria. However, mosquitoes caused the biggest challenge. Many said that, because of mosquitoes, they had to leave work well before dark, and were forced to go to sleep under the mosquito nets early. Women had to leave work even earlier as they had to also prepare meals, and all these had to be completed before it got dark. They also emphasized that malaria was among the biggest problems that reduced productivity at the farms, as often people had to leave work to go back to the towns to seek medical treatment, or back home until they got better. This slowed down the work in the farms, and sometimes forced them to stay for longer period of times. Below are examples of comments from the participants.

- The main challenge is suffering from malaria which affects our ability to be productive, (50 year old, female, Igumbiro village).
- When we are infected, we normally go home for treatment then return to the farm, (43 year old female, Lupiro village).
- When we are at the farm, my children and I put on long clothes that cover us to the feet from eighteen hours in the evening and we sleep under mosquito nets, (43 year old female, Lupiro village).
- We try to fan them (mosquitoes) off but they keep on biting us, so we just go on with our chores until it’s time to go to bed, then we sleep under the mosquito nets, (36 year old female, Lupiro village).

### Views of the migratory farmers regarding effectiveness of their traditional *Shamba* huts in preventing mosquito entrance

The participants said that their traditional huts did not provide adequate protection against mosquitoes. These huts had many holes through which mosquitoes freely could enter and leave (Fig. 1b, c). Some participants said that they put mosquito nets over, rather than inside their *Shamba* huts, hoping to prevent mosquitoes from getting in, but the nets get torn, hence having many holes through which the mosquitoes get through. They also said that they huts are full of mosquitoes both during the day and night. Here are some quotes from the participants:

- We use grass to roof our shelters or sometimes a piece of Khanga (a type of cloth mostly used by women to wrap around their waists, while perfoming different chores) to enclose the house, which is not enough. We only trust the mosquito nets for protection, (36 year old female, Lupiro village).
- I have the same problem; the mosquito nets have holes hence the mosquitoes enter inside, (40 year old female, Igumbiro village).
- The mosquito nets we are currently using we put them on top of our huts sometimes they are torn by stick and allow mosquito passage, (50 year old female, Igumiro village).

## Discussion

Many previous studies have reported that despite high densities of mosquito vectors in rice growing areas, pathogen transmission is often lower, partly because of: (a) the lower human densities in these sites, (b) the high proportions of mosquito feeding on non-human blood sources, (c) lower pathogen prevalence in the mosquito populations, and occasionally, (d) the higher living standards among rice growers [34, 35]. In an earlier study conducted by Hetzel et al. in south-eastern Tanzania, the authors reported that fever cases were similar people staying at home and those spending long periods of time in the rice fields, and that there was no excess fever risk associated with this practice [22]. Hetzel et al. followed 100 households for 6 months, each month asking about the whereabouts of family members, whether any of the family members had experienced fever cases in previous 2 weeks, and what kinds of treatments they sought. They however did not conduct any parasitological or entomological assessments to assess actual risk of malaria infection, and it is likely that any differences may have been attenuated at this time given malaria transmission rates in the area were extremely high and likely saturated, with individual community members receiving up to 400 infectious mosquito bites per person per year in those years [21, 36]. Other studies however reported higher malaria episodes in the agricultural than non-agricultural areas [37], and in rice irrigation sites compared to places where irrigation was interrupted [38], suggesting that any relationships between agriculture and mosquito-borne pathogen transmission may vary immensely between sites.

It is likely that in residual malaria systems, where transmission has been reduced significantly, and where malaria is unstable, the presence of migratory farmers, who may harbor parasites in their bodies for long periods without treatment, and are far from health facilities becomes a major concern for elimination efforts. Ijumba and Lindsay [39] referred to this phenomenon as the “paddies paradox”, and explained that higher vector densities in rice farming communities can lead to increased malaria in unstable transmission sites where people have little or no immunity to malaria parasites, such as in the African highlands and desert fringes, but that such effects would not be obvious in most stable transmission systems.

In this study, equal numbers of female mosquitoes were caught indoors of main houses and *Shamba* huts. This is probably due to higher biomass of individuals within the villages as compared to the farms, which leads to increased density of mosquitoes [34], but possibly also because the collections were done outside the peak rainy seasons. However, laboratory analysis of *Anopheles* mosquitoes from both the main and *Shamba* hut did not detect any *Plasmodium* sporozoites, thus were unable to determine where there were higher malaria transmission levels between the main houses and *Shamba* huts. The laboratory findings support those of Hetzel et al. [22] and, therefore, suggests that it is mostly nuisance bites that the rice farmers experience while in the rice fields. On the other hand, it may be that other mosquito-borne pathogens, possibly including arboviruses, transmitted by a variety of mosquito species, remain predominant in these rice fields. Although no difference in risk of malaria infections was seen between the main and *Shamba* huts, it is clear that as the heterogeneity of malaria transmission is constantly changing, there is a need to improve the current housing structures being used by the farmers.

The burden of malaria in many African communities has indeed drastically reduced in the past 15 years due to life-saving interventions like LLINS IRS and improved diagnosis and treatment, aided by urbanization, improved living standards and better health care. LLINs and IRS combined, have contributed about 78% of all gains accrued since 2000 [40]. In rural south eastern Tanzania, where long-lasting insecticidal bed nets have been widely used, malaria prevalence reduced by more than 60% since 2001, low-level transmission still persists [12]. Amid these declines, malaria epidemiology is also increasingly stratified [41], with geographically distinct pockets of high transmission [42], or demographically distinct sub-populations, such as forest workers and rice farmers [43]. Previous assessments have demonstrated effects of such occupation-related exposures and how they contribute to overall transmission dynamics of common pathogens including malaria [44]. This is a particularly common occurrence in south-east Asia where nearly two-thirds of malaria cases in some places occur in the forest or forest fringe areas and where the highest risk groups include internal migrants, subsistence farmers in the forest and forest fringes and forest workers, as in Myanmar [45] or in the dry season inside the forest as in Thailand [46].

In rural south eastern Tanzania, there is therefore a dire need of improving housing structures of these migratory farmers, particularly because these farmers not only shift to the farms for periods as long as 6 months or more, but also because they go together with their young children. Below is an excerpt from one of the female interviewees:

Interviewer: Okay, thanks. And when you shift to the farm how long do you stay?
Respondent: January to July
Interviewer: … If you go the farm do you go with your young ones?
Respondent: I take with me the youngest, those who are going to school remain here (home) until Friday then they come there (to the farm)
Interviewer: How old is the youngest?
Respondent: Three years old
Interviewer: So will you be going with him/her to the farm until he/she starts schooling or?
Respondent: Yes, I will be going with him until he starts going to school. Then he will be remaining at home

The portable mosquito-proof hut prototype, i.e. the *Swai* hut, might be a plausible solution for these farmers. The prototype has so far shown full protection against mosquitoes in both trails in semi-field and field settings. The design makes it a better housing structure than the semi open improvised structures currently being used by the migratory farmers in the rice fields, and confirms findings from studies done showing that improved housing as a means to reduce malaria cases [8, 19, 47–49].

Although the *Swai* hut proved to be 100% effective in controlling mosquito entry, it still had some limitations including production cost i.e. $1460.38, which was too high, need for stable but raised surfaces, inability to cook inside the huts due to fire risk, and the fact that the huts are protective only when the users are inside them. To ensure the product is more consumers friendly both in its price and use, the following can be done: Using an alternative fabric that would cost lower than the expensive ribstop canvas, which would significantly reduce the overall costs by between half and two-thirds. Also, having the *Swai* hut produced at a commercial level with lighter metallic frames or more readily available wooden frames, other than the steel bars we used for this proof-of-principle prototype, will further reduce the overall cost substantially while making it more portable to the user. Adding stabilizing wires/ropes similar to those of tents at each corner of the *Swai* hut, would increase stability when the hut is on raised surfaces. Coating the UV resistant netting material with fire retardants would also help with reducing fire risks if one decides to cook inside. Additionally, having a veranda made of UV resistant netting coated with boric acid extending from the main body, would not only allow users to be able to cook with minimal risk of fire burning the hut, but they would also have a place to relax and story tell without worrying about mosquito bites.

The authors expect that at optimum production, a portable mosquito-proof hut for two persons could be produced for as low as 210 US$ per unit and would last at least 3 years without replacement, thus effectively providing protection for <35 US$ per person per year.

The tests described here demonstrate that such simple innovations could be most readily applicable for protecting disenfranchised communities, such as these migratory farmers, but possibly also others like forest workers and pastoralists.

## Conclusion

Migratory rice farmers in the residual transmission settings in rural south-eastern Tanzania do not experience more mosquito bites than the general population, but, like the rest of the population, these farmers also engage in various risk-prone outdoor activities that expose them to excessive outdoor-biting by potentially infectious mosquitoes. While this study could not confirm higher malaria transmission rates in the *Shamba* huts than in the main houses, their reduced access to organized health care, inability to effectively use available mosquito control methods like LLINs and the higher mosquito blood-feeding rates in these huts, make these itinerant households more vulnerable than the general population. The newly developed and tested *Swai* hut prototype offered full protection against malaria mosquitoes both in the field and SFS, and community members readily accepted and like it. Changes in house structure can result in reduction of indoor mosquito density but also allow proper use of interventions like ITNs. This portable mosquito-proof hut therefore demonstrates how improving house structure can limit the entry of mosquitoes and reduce biting by nuisance and disease-transmitting mosquitoes. The *Swai* hut is also an example of how simple innovations such as this could be used to expand protection for disenfranchised communities like the migratory farmers in rural south-eastern Tanzania, but possibly also forest workers, miners and pastoralist communities. Further improvements and testing of different designs made from different fabrics is necessary to lower prices without compromising long-term protective efficacy against mosquito-borne infections.
